# From glucose sensing to exocytosis: takes from maturity onset diabetes of the young

**DOI:** 10.3389/fendo.2023.1188301

**Published:** 2023-05-15

**Authors:** Sama Samadli, Qiaoli Zhou, Bixia Zheng, Wei Gu, Aihua Zhang

**Affiliations:** ^1^ Nanjing Key Laboratory of Pediatrics, Children’s Hospital of Nanjing Medical University, Nanjing, China; ^2^ Department of Pediatric Diseases II, Azerbaijan Medical University, Baku, Azerbaijan; ^3^ Department of Endocrinology, Children’s Hospital of Nanjing Medical University, Nanjing, China

**Keywords:** MODY, maturity onset diabetes of the young, glucokinase, channelopathy, unfolded protein response, transcription factors, carboxy ester lipase

## Abstract

Monogenic diabetes gave us simplified models of complex molecular processes occurring within β-cells, which allowed to explore the roles of numerous proteins from single protein perspective. Constellation of characteristic phenotypic features and wide application of genetic sequencing techniques to clinical practice, made the major form of monogenic diabetes – the Maturity Onset Diabetes of the Young to be distinguishable from type 1, type 2 as well as neonatal diabetes mellitus and understanding underlying molecular events for each type of MODY contributed to the advancements of antidiabetic therapy and stem cell research tremendously. The functional analysis of MODY-causing proteins in diabetes development, not only provided better care for patients suffering from diabetes, but also enriched our comprehension regarding the universal cellular processes including transcriptional and translational regulation, behavior of ion channels and transporters, cargo trafficking, exocytosis. In this review, we will overview structure and function of MODY-causing proteins, alterations in a particular protein arising from the deleterious mutations to the corresponding gene and their consequences, and translation of this knowledge into new treatment strategies.

## Introduction

The incidence of Maturity Onset Diabetes of the Young (MODY) is accounted for 1-2% of total diabetes cases and is expected to rise due to increasing awareness and better identification from more prevalent forms of diabetes by endocrinologists (1). Clinically, MODY patients present with following features: a strong family history of diabetes with an apparent autosomal-dominant inheritance, onset of diabetes before 25 years of age in at least one generation of the same family, sustained endogenous insulin production without insulin resistance and absence of β cell-specific autoantibodies (2). However, it often shares common features with the frequently seen forms of diabetes, which in turn creates challenges for making diagnosis. Luckily, wide application of genetic sequencing methods in clinical settings helps to avoid pitfalls in most cases (3). Despite of the fact that genetic testing methods are gold standard in the discovery of MODY-causing genes, the etiology of MODYX cases are yet to be identified (4). Besides, by means of more sensitive approaches, the later genotype-phenotype correlation studies revealed that some genes from the previous established list of 14 MODY genes should not be regarded as causative for MODY (5, 6).

**Figure 1:**
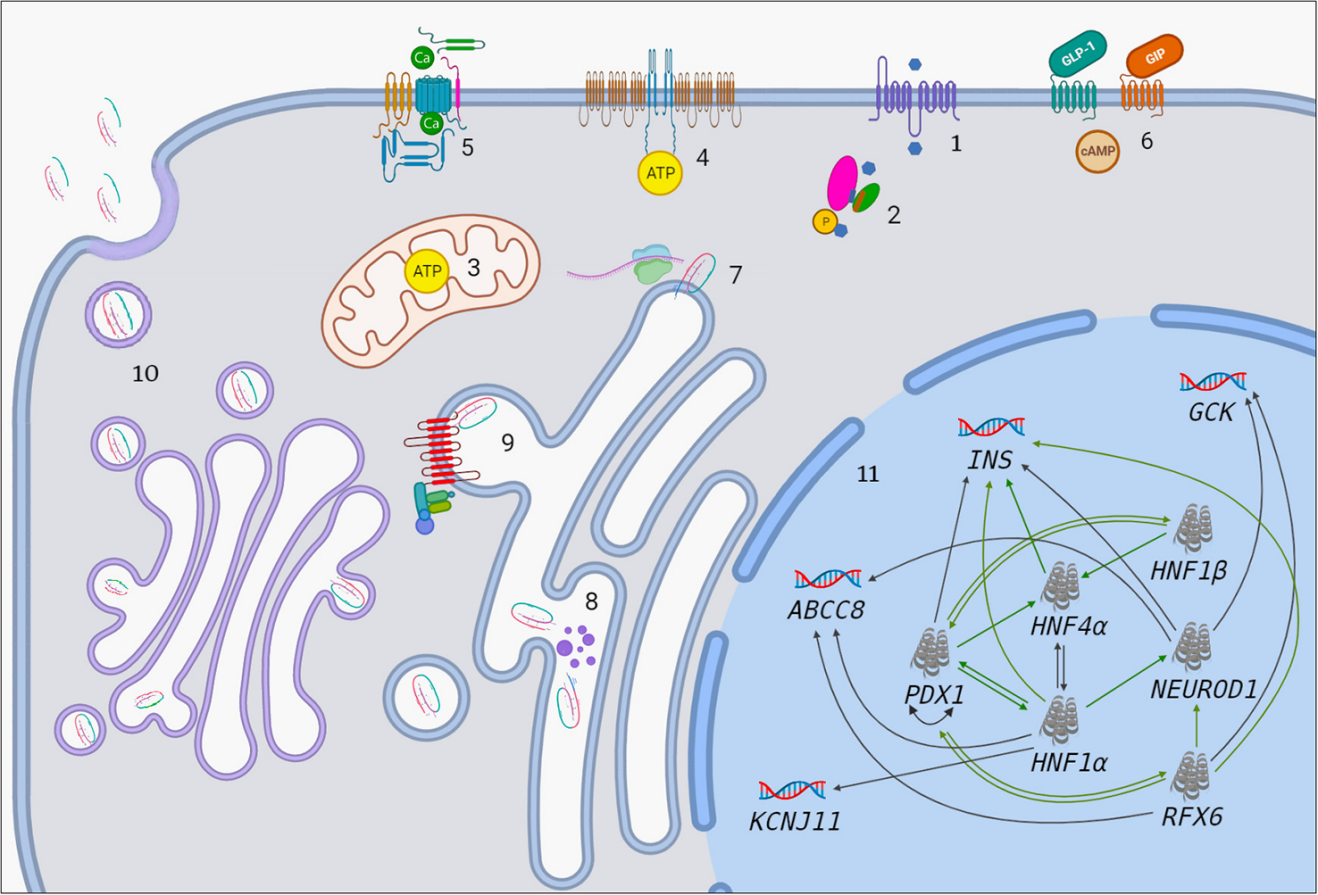
The association between MODY-causing proteins. 1. Influx of glucose to β-cell through GLUT-2. Blue hexagon represents glucose. 2. Transition of glucose to glucose-6-phosphate by GCK enzyme. Pink oval, green oval, purple cylinder and orange cylinder represent large domain, small domain, glucose-binding site and ATP-binding site of GCK, respectively. 3. Increased ATP production in mitochondria after the entry of G6P to Krebs cycle. 4. Closure of ATP-sensitive potassium channels. 5. Opening of voltage-gated calcium channels. 6. GIP and GLP-1 bind to their receptors and initiate number of events which result in enhanced insulin synthesis and secretion (There is apparent link between GIP/GLP-1 and MODY-causing molecules such as PDX1, RFX6. Molecular basis of this is unknown but it has therapeutic implications). 7. Preproinsulin biosynthesis in cytosol. 8. Preproinsulin processing in ER by endopeptidases (purple dots) and subsequent cleavage of signal peptide. 9. ER-membrane located wolframin interacts with proinsulin in one end and COP-II proteins in other end and helps formation of COP-II-coated proinsulin vesicles. 10. Proinsulin processing in Golgi apparatus, final vesicle formation, release of C-peptide from insulin in Golgi apparatus and inside the vesicles and exocytosis. 11. Transcriptional regulation in the nucleus. Black arrows indicate regulation of a given gene by the relevant TF in mature β-cell. Green arrows indicate that a given protein has binding site for the relevant TF and/or had been regulated by it at some point of pancreatic development.

Understanding molecular basis of MODY gave comprehensive knowledge concerning the physiological processes in functional β-cells during glucose-stimulated insulin secretion (GSIS) and insulin synthesis. Glucose sensing of β-cells is the first step in maintaining normoglycemia, characterized by transfer of glucose – which is entered into the cell through cell membrane related glucose-transporter-2 protein (GLUT-2) – to glucose-6-phosphate by glucokinase (GCK). Glucose-6-phosphate enters the Krebs cycle inside the mitochondria and results in enhanced production of ATP. Increased level of ATP induces closure of K_ATP_ channels and depolarize the cell membrane to approximately -30 mV. This in turn leads to opening of L-type voltage-gated calcium channels, increased entry of Ca^2+^ ions into the cell and subsequent release of insulin (7). Glucose homeostasis of an organism is also maintained by proper and adequate insulin synthesis. Insulin is translated in the cytosol as 110 amino acids-length preproinsulin, and then undergoes posttranslational modification to form proinsulin in the endoplasmic reticulum. Following transitioning of proinsulin to the Golgi apparatus culminates in formation of secretory granules composing of Zn^2+^ ion coupled insulin, proinsulin, C-peptide, amylin and regulatory proteins which are responsible for completion of “ready to use” insulin maturation (8, 9). Key steps of insulin biosynthesis and secretion are regulated by transcriptional network constituting of hierarchical, auto- and inter-regulatory complex of transcription factors (TFs), some of which involved in the pathogenesis of MODY (10) (**Figure 1**).

Accumulated knowledge regarding the molecular events in the basis of MODY allows better management strategies and resultant improved quality of life in MODY patients (11). Nevertheless, increased understanding of core molecular processes offers benefits beyond the high-quality care for MODY patients. Considering the fact that TFs such as PDX1, NEUROD1, HNF family proteins, play a crucial role in the development and differentiation of embryogenic pancreas and in the maintenance of functional mature islet cells (**Table 1**), the use of these key regulatory proteins in the establishment of insulin-producing β-cells from stem cells paved the way for radical treatment of insulin-dependent forms of diabetes mellitus (12, 13). In this review, we will overview structure and function of MODY-causing proteins, alterations in a particular protein arising from the deleterious mutations to the corresponding gene and their consequences, and translation of this knowledge into new treatment strategies.

**Table 1:** The characteristics of MODY-causing proteins.

Protein name	Amino acid length	Molecular weight	Functions in pancreas	Phenotype		Treatment considerations
				Homozygosity	Heterozygosity	
HNF4α or TCF14	452 (isoform-7)	50 kDa	Foregut differentiation; insulin synthesis and secretion in mature β-cells	Not reported	MODY1; often accompanied with dyslipidemia, transient neonatal hypoglycemia and macrosomia	Low-dose SUs or DPP-4 inhibitors in uncomplicated cases
GCK or Hexokinase-4	465 (isoform-1)	52 kDa	Glucose-sensing and phosphorylating enzyme	Inactivating mutations cause PNDM	Activating mutations cause HH; while inactivating mutations lead to MODY2	Diet and exercise are sufficient in majority of MODY cases. GCK activators are promising for T2DM
HNF1α or TCF1	631	76 kDa	Foregut differentiation; insulin synthesis and secretion in mature β-cells	Not reported	MODY3; often accompanied with higher HDL-cholesterol and lower LDL-cholesterol levels, and transient neonatal hypoglycemia and macrosomia	Low-dose SUs or DPP-4 inhibitors in uncomplicated cases. MODY3 patients may show exaggerated response to SUs
PDX1 or IPF1 or STF1 or IDX1 or GSF or IUF1	283	30.8 kDa	Master regulator of pancreas formation, differentiation and maturation of islet cells; insulin synthesis and secretion in mature β-cells	Pancreatic agenesis or ND	MODY4 or gestational diabetes	From diet to insulin replacement. PDX1 mutations are linked to lower levels of incretins and DPP-4 inhibitors show efficacy in heterozygous cases
HNF1β or TCF2	557 (isoform-A)	61 kDa	Pancreatic development since primitive gut stage; function in mature β-cells is unknown	Not reported	MODY5 or Renal-cysts-diabetes syndrome	Mainly insulin replacement
NEUROD1 or BETA2	356	39 kDa	Normal endocrine cell development and insulin synthesis and secretion in mature β-cells	PNDM with more severe neurological phenotype	MODY6 characterized with ketoacidosis-prone diabetes with microvascular sequelae and neurological abnormalities	From diet to insulin replacement
CEL or BSDL	753	74 kDa	Pancreatic exocrine enzyme secreted to digestive tract	No report on diabetes	MODY8 characterized with pancreatic exocrine insufficiency with DM	Currently, insulin replacement
INS	51	5.8 kDa	Hormone that regulates glucose metabolism in organism	PNDM	MODY10	Currently, insulin replacement. UPR targeting agents might be beneficial
SUR or SUR1	1581 (isoform-1)	177 kDa	β-cell excitation as a part of ATP-sensitive K+ channel	Inactivating mutations cause HH, activating mutations lead to DEND syndrome, TNDM, PNDM or MODY12	Inactivating mutations cause HH, activating mutations lead to DEND syndrome, TNDM, PNDM or MODY12	High-dose SUs for patients harboring activating mutations
Kir6.2	390 (isoform-1)	44 kDa	β-cell excitation as a part of ATP-sensitive K+ channel	Inactivating mutations cause HH, activating mutations lead to DEND syndrome, TNDM, PNDM or MODY13	Inactivating mutations cause HH, activating mutations lead to DEND syndrome, TNDM, PNDM or MODY13	High-dose SUs for patients harboring activating mutations
Wolframin	890 (isoform-1)	100 kDa	Cargo receptor involved in vesicle formation and exocytosis	Wolfram syndrome	Wolfram syndrome or MODY	Currently, insulin replacement. UPR targeting agents might be beneficial
RFX6	928	102 kDa	Formation of dorsal pancreatic bud and insulin synthesis and secretion in mature β-cells	Mitchell-Riley syndrome	Non-autoimmune, late-onset diabetes	The association between RFX6 mutations and lower levels of incretins supports treatment with DPP-4 inhibitors

## Impaired glucose sensing

### *GCK* mutations (MODY2)

GCK is a glycolytic enzyme initiates glucose utilization and acts as a glucose-sensor through controlling glucose phosphorylation in β-cells (14). Heterozygous inactivating mutations in *GCK* gene are responsible for MODY2, which constitutes the highest proportion of all MODY cases (15). Heterozygous activating *GCK* mutations lead to HH, while inactivating mutations in both alleles cause permanent neonatal diabetes occurring in the first 6 months of life, explaining the vital role of GCK enzyme in glucose homeostasis (**Table 1**) (16).

**Figure 2:**
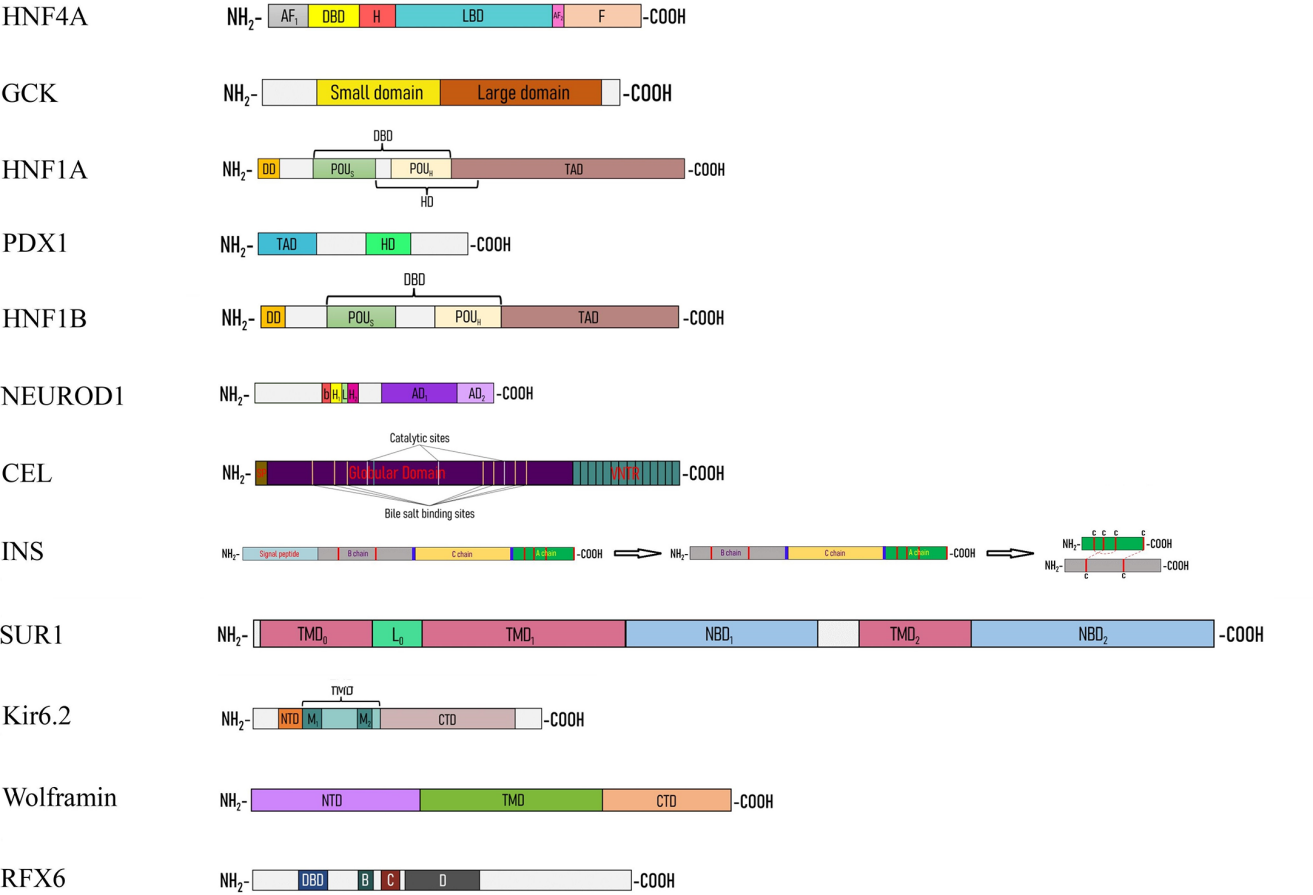
Linear cartoon structure of MODY-causing proteins. *AF1(2)* – activation function 1(2), *bHLH* – basic helix-loop-helix, *CTD* – C-terminal domain, *DBD* – DNA-binding domain, *DD* – dimerization domain, *H* – hinge, *HD* – homeodomain, *L_0_* – loop, *LBD* – ligand binding domain, *NBD* – nucleotide binding domain, *NTD* – N-terminal domain, *TAD* – transactivation domain, *TMD* – transmembrane domain.

Pancreatic glucokinase is composed of large and small domains separated by glucose binding active site, also known as deep cleft (**Figure 2**) (17). Lower affinity for glucose in its passive super-open form and lack of inhibition by the end product are their main properties, allowing to exhibit its rate-limiting catalytic activity. According to the mnemonic model hypothesis (18), under the influence of 5 mM or higher glucose concentration, super-open form transforms into open form, which directly binds to ATP and changes into closed form. Ongoing influx of glucose, sustains the transition between open and closed forms and this step is termed as fast cycle. On the contrary, lower level of glucose makes the molecule to regain its super-open form in a slow cycle fashion (19, 20). To date, more than 600 missense and nonsense mutations were discovered in the *GCK* gene of families suffering from MODY2 which alter glucose and ATP-binding abilities of the enzyme through directly impairing its kinetic parameters, or interfering with either structural stability or posttranslational regulation of the protein (16, 21). Certain missense mutations impede kinetic activity of GCK enzyme *via* stabilizing its inactive form or causing electrostatic repulsion of particles within active (closed) configuration (22). On the other hand, some mutations are associated with thermal instability affecting the three-dimensional structure of GCK (23). Functional analysis found that mutations remote to the active site of GCK enzyme prevented GCK-containing granules translocate to the cytoplasm by unidentified posttranslational regulator in insulin-producing pancreatic cells (24). Lastly, a rare mutation in the *GCK* β-cell promoter revealed Sp1 as one of the human transcription factors, although it is considered that inclusion of promoter analysis to routine sequencing methods would yield higher detection of such mutations (25).

Taking advantage of central role of GCK in glucose homeostasis, its activators – which are able to bind to the allosteric site, change kinetic parameters of GCK, and render the molecule more sensitive to glucose – have gained a lot of attention for better management of Type 2 Diabetes Mellitus (T2DM). While majority of these agents failed to show sustained glycemic control (26), brand-new drug – dorzagliatin passed phase 3 trial successfully and is hoped to fulfill the need for more effective hypoglycemic agent with less undesirable events (27). Some MODY2 mutations may decrease efficient targeting of GCK by these agents (28). Nevertheless, since MODY2 mutations cause GCK to be hyposensitive rather than insensitive to glucose, most MODY2 patients have mild fasting hyperglycemia and lower chance of complications and they achieve good control over glucose levels for several years with only lifestyle modifications (16).

## Channelopathies

### K_ATP_ channelopathies

K_ATP_ channel are composed of four pore-forming Potassium inward rectifier 6.2 (Kir6.2) subunits and four regulatory sulfonylurea receptor 1 (SUR1) subunits, which are encoded by two neighboring genes: *ABCC8* and *KCNJ11*, respectively (29). SUR-1 shows both stimulatory and inhibitory effect on Kir6.2 through sensitizing or desensitizing Kir6.2 to ATP, which are best represented by the impact of activating and inactivating mutations on this channel. Gain-of-function mutations of these genes cause DEND syndrome, permanent or transient NDM, MODY or T2DM susceptibility depending on the degree of metabolic impairment within β-cells and other cell types (30). Loss-of-function mutations, on the other hand, produce HH attributed to either loss of channels on the cell surface or decreased channel activity (**Table 1**) (31). Compelling amount of knowledge regarding the molecular basis of K_ATP_ channelopathies extrapolated from the mutational analysis of NDM or HH. MODY emerges as a mild form of NDM due to lesser increase in K_ATP_ channel current (30) or as a consequence of exhausted β-cells in HH (32).

#### *ABCC8* mutations (MODY12)

SUR1 subunit comprised of a functionally active five-helix transmembrane domain 0 (TMD0), six-helix TMD1 and TMD2, a cytosolic loop (L0) between TMD0 and TMD1 and cytosolic nucleotide-binding domains NBD1 and NBD2 (**Figure 2**). NBDs act as cytosolic sensors and channel openers and are modified by Mg-nucleotides to create 2 nucleotide-binding sites. Nucleotide-binding sites 1 and 2 are formed by Walker A and B motifs of one NBD with the signature sequence located between Walker A and B of the head-to-tail aligned opposite NBD. Dimerization of nucleotide-binding sites is translated into conformational change of the TMD1 and TMD2, then subsequent transformation of Lasso motif of the L0 loop and TMD0, and eventually, clockwise rotation of Kir6.2 cytoplasmic domains to open the pore (33). Substitutions in the linker region is associated with impaired transduction, rather than altered Mg-nucleotide-binding and hydrolysis. This indicates NBDs interact with TMDs *via* linker region (34). Mutations that elicit the impairment of Mg-nucleotide-binding and/or catalytic hydrolyzation at the nucleotide-binding sites abolish stimulatory effect of SUR1 on Kir6.2 and result in closure of channels (35, 36). On the other hand, activating mutations of NBD1 and NBD2 hyperpolarize pancreatic β-cells and lead to hyperglycemia owing to reduced K_ATP_ channel inhibition caused by slow deactivation of Mg-ADP from its bound state (37–39), although nucleotide-binding sites show asymmetry with their distinct preference to binding and hydrolysis of nucleotides (40). Interestingly, the effects of the interaction of Mg-nucleotides with SUR1 on Kir6.2 are best represented in the reports, which same residues cause opposing phenotypes – either diabetes or hyperinsulinism depending on the amino acid substitution (41). NDM mutations at TMDs are characterized by disrupting the transduction and abrogating the stimulatory effect of NBDs on Kir6.2 (42, 43). Moreover, unusual phenotype of alternating hypoglycemia and hyperglycemia are reported in a patient with homozygous *ABCC8* mutation, even though heterozygous inheritance of the same mutation caused severe trafficking defect (44). Above reports indicate that slight differences in the molecular mechanism of *ABCC8* mutations manifest with completely distinct clinical picture.

#### *KCNJ11* mutations (MODY13)

Kir6.2 subunit is formed by N- and C-terminal cytoplasmic domains, TMD containing M1 and M2, between which pore loop and selectivity filter locate, and tether helix linking TMD to CTD (**Figure 2**). It possesses ATP-binding domain which plays crucial role in coupling channel inhibition to metabolic activation (45). Disrupted ATP-binding and hydrolysis, altered intersubunit interactions and morphology of the channel, transduction abnormalities, deranged allosteric regulation of ATP sensitivity and disconnection with SUR1 interfere with proper functioning of Kir6.2 and lead to imbalance between K_ATP_ inhibition and high glucose levels (46–51). ATP interacts with Kir6.2 as proposed: G334 residue of the solvent-exposed helical segment with α-phosphate of ATP, K185 residue of the C-terminal β-sheet with both α- and β-phosphates and R50 residue of the N-terminal peptide with γ-phosphate. ATP brings NTD and CTD of Kir6.2 subunits with L0 of SUR1 together and locks the channel in closed state (45). The mutations in these residues are well documented in patients suffering from ND and DEND syndrome (49). On the other hand, mutations which are located far from ATP-binding site could also prevent normal ATP-binding and hydrolysis. Some substitutions to opposite charged amino acid increase steric repulsion between two adjacent ATP-binding pocket monomers (50), while others derange ATP-binding site, which make them less flexible for ATP to be able to fit (47). Impaired transduction from ATP-binding pocket to gate is another mechanism for Kir6.2 related diabetes, mostly associated with the mutations of slide helix (48). Structural integrity is key for effective channel behavior, which could be disrupted by the mutations through compression of the ion conducting part of the channel or halted intersubunit interactions (50). Mutations that cause disconnection between Kir6.2 and SUR1 decrease ATP sensitivity of the channel, even though intrinsic gating properties are unchanged (46). In addition to nucleotides, other allosteric regulators such as PIP_2_ also play major role in gating. Hypersensitivity to PIP_2_ due to cysteine substitutions increases open probability of K_ATP_ *via* enhancing S-palmitoylation (51). Furthermore, understanding underlying molecular mechanisms of channelopathies gives clue to why identical mutations show phenotypic heterogeneity. In MODY13 patients, late occurrence of the disease is not just accounted for lesser half maximal inhibitory concentration of ATP (52) and also additional trafficking defect of the channel which compensate for the increased open probability (53).

Molecular basis of MODY12 and MODY13 warranted to switch treatment option from insulin to sulfonylurea, which has 90% success rate (54, 55). Cryo-EM structure analysis revealed that glibenclamide binds to its binding site, which is formed by helices 6-8,11 of TMD1 and helices 16,17 of TMD2 of SUR1, and stabilize the interactions between N-terminus of Kir6.2 and L0 and TMD0 of SUR1 to prevent opening of the channel (45). Treatment failure is partly explained by the type of the mutation (56). It is obvious that mutations causing morphological changes in pore-forming subunit (50) or abnormal SUR1-Kir6.2 transduction (57) render the channel insensitive to SUs. There is no report on the SU binding site defect related treatment failure.

## Impaired insulin trafficking

### *INS* mutations (MODY10)

Mature insulin (**Table 1**) is a small molecule composed of A and B chains linked with 3 disulfide bonds. After synthesizing in the cytosol, preproinsulin is subjected to SRP facilitated translocation to ER, where it is cleaved to form proinsulin. Proinsulin in turn is liberated from C peptide *via* the recruitment of ER chaperons (**Figures 1**, **2**) (58). Dominant and recessive mutations to the *INS* gene give rise to wide range of phenotypes varying from late-onset mild hyperglycemia to severe neonatal diabetes. Type of mutation is the main determinant of clinical phenotype (59) and as the best characterized monogenic mutations, analysis of insulin substitutes provided with broad knowledge regarding the insulin biosynthesis, action, and tight control of all these processes.

Cysteine substitutions interrupting formation of the disulfide bonds (B7-A7, B19-A20 and A6-A11) are classic examples of protein misfolding which lead to: 1) ER retention of both mutant and wild-type protein due to inappropriate interactions between unpaired cysteine of mutant protein and wild-type molecule (60); 2) impairment of β-cell proliferation by subsequent ER stress (61). Besides, any substitution to cysteine outside of the disulfide bonds produce similar effect (62). However, non-cysteine substitutions are also able to perturb normal folding of proinsulin (63). Preproinsulin recognition by signal peptidases and translocation of it to ER are initiating events in insulin maturation, which are disrupted by mutations to the signal peptide of preproinsulin (64). In order to fold and deliver properly to Golgi apparatus, proinsulin must be cleaved by endopeptidases at the junctions between C-peptide and each of the chain (58). Notably, mutations to these junctions underlie mild and late-onset diabetes accompanied by hyperproinsulinemia (65). Furthermore, some mutations may also interfere with insulin binding to its receptor. Resultant hyperinsulinemia is explained by disrupted degradation of insulin which fails to uptake by hepatocytes (65). Apart from the coding regions, the mutations affecting regulatory sequences are also described. Mutations to the promoter region of *INS* gene preventing the TFs to bind to its cis-elements; substitutions at the start codon abolishing translation; and alterations at the untranslated sites of mRNA sensitizing the molecule to RNA decay – all fall into this category (66).

As expected, MODY10 predominantly is managed by insulin injections (67), albeit understanding of underlying molecular mechanisms of *INS* mutations offered new perspectives for better therapeutic options. Regarding this, rescuing trapped wild-type proinsulin from ER through either degradation of mutant molecule or acceleration of oxidative folding is suggested by some laboratories (68, 69).

### *WFS1* mutations (Newly proposed MODY type)

Wolframin (**Table 1**) is a cargo-protein receptor, located in the ER membrane and contains two hydrophilic N- and C-terminal domains and the hydrophobic transmembrane domain between them (**Figure 2**) (70). Patients bearing homozygous and compound heterozygous mutations of *WFS1* gene present with different combinations of young-onset diabetes mellitus, diabetes insipidus, optic atrophy and hearing loss, which are classical features of Wolfram syndrome (71). Wide application of sequencing techniques in diverse populations brought to light significant number of heterozygous *WFS1* gene mutations contributing to MODY phenotype (72–75), although homozygosity is not always exception in MODY incidence (76). The knock-out mice generated with CRISPR-Cas9 technique highlighted the role of wolframin in proinsulin trafficking and vesicle formation. The study showed that NTD mutations disrupt interactions with Coat Protein Complex II (COPII) vesicle subunits, while CTD mutations interrupt wolframin-proinsulin bonds, both of which activated unfolded protein response pathway (70).

In the recent years, induced pluripotent stem cell models derived from MODY patients, have gained a lot of attention as an exciting tool to deepen the understanding of cellular and molecular processes of mature and developing β-cells in healthy and pathological states of pancreas, as well as to introduce new treatment strategies (77). β-cell iPSC models derived from patients with Wolfram syndrome helped to demonstrate that curative treatments such as CRISPR-Cas9 correction of a mutant gene or chemical chaperons targeting UPR pathways are not far-fetched for patients suffering from variety of ER stress causing diseases (78, 79).

## Impaired transcriptional regulation

**Figure 3:**
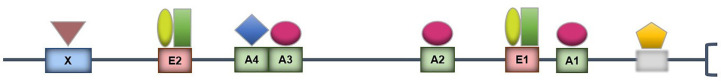
Schematic illustration of the binding sites of insulin promoter for MODY-related transcription factors. 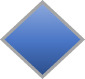
HNF1α 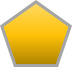
HNF4α 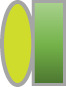
NEUROD1/E47 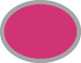
PDX1 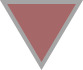
RFX6.

TFs are the group of proteins bind to cis-acting elements of the promoter of particular genes and regulate their transcriptional activity. MODY-related TFs (*PDX1, NEUROD1, HNF1α, HNF4α, HNF1β, RFX6*) are indispensable for the development of pancreas, β-cell identity and function of mature β-cells (**Table 1**) (80) and they all have direct binding sites at the insulin promoter (**Figure 3**) (81–84). Recently, detailed in silico analysis of available published data revealed that two TF genes, namely *KLF11* and *PAX4* – which previously enlisted as MODY-causing proteins – can not be included in the MODY list due to lack of co-segregation with diabetes and higher rate of same mutant genes in healthy population in addition to two other non-TF genes –*BLK* and *APPL1* (5, 6). Thus, accurate co-segregation analysis of genetic test results is crucial to avoid misinterpretations.

### *HNF4α* mutations (MODY1)

HNF4α (**Table 1**) has a N-terminal AF1 domain that shows ligand-independent transactivation ability, a highly-conserved DNA-binding domain containing zinc-finger motif, a lipophilic ligand-binding domain, and a regulatory F domain (**Figure 2**). The domains are interrelated in such a way that the remote residues may have allosteric modulation over other sites owing to domain-domain interactions (85). Hyperglycemia in MODY1 is frequently accompanied by dyslipidemia and preceding transient neonatal diabetes with macrosomia (86). The latest report on MODY3 patient-derived iPSC lines solved the long debate of biphasic nature of disease by determining direct binding sites of *ABCC8* and *KCNJ11* genes for HNF4α (87). Detailed analysis of biphasic MODY1-causing LBD mutations revealed that dimerization of HNF4α allows it to conform to more structurally stable form and to expose more hydrophobic sites at the surface so that LBD may able to be occupied by its lipid ligands to the fullest extent (88). In DNA-binding motif, negatively charged amino acid substitutions for serine residues result in both lesser DNA-binding and transcriptional activity. Additionally, these findings also discovered the inhibitory effect of PKA-phosphorylation on DNA-binding and transactivation of HNF4α (89). While most single amino acid substitutions lead to structural and/or binding defect, some of them induce proteolytic degradation which turn the protein into a smaller truncated form (90). In addition to coding region, MODY1 is also originates from the mutations in the pancreas-specific P2 promoter (91). Functional studies found the binding sites of the P2 promoter for the TFs including HNF1α, HNF1β, PDX1 (91) along with direct binding of HNF4α to the *INS*, and *HNF1α* promoters (82, 92, 93), all of which indicate that β-cell function is controlled by complex and multifaceted hierarchical and interregulatory network.

MODY1-derived iPSC lines provided with model that represents the early stages of formation of human hepatopancreatic tissues. According to these models, insufficient amount of HNF4α downregulates foregut-specifying genes while upregulating hindgut markers and impacts normal foregut endodermal cell fate acquisition (94).

### *HNF1α* mutations (MODY3)

HNF1α (**Table 1**) is a homeodomain-containing transcription factor of *HNF* family and it consists of a dimerization domain, a DNA-binding domain with POU-like and homeodomain-like motifs, and a transactivation domain in an order from amino-terminus to carboxy-terminus (**Figure 2**) (95). HNF1α binds to promoters of different genes including *PDX1, INS, GLUT-2* and controls glucose sensing, mitochondrial metabolism, insulin secretion and exocytosis in β-cells (96–99). MODY3 mutations are dominant loss-of-function mutations (100) that cause the clinical phenotype identical to MODY1, if higher levels of HDL-cholesterol are excepted (86). Mutations affecting dimerization domain are characterized with more severe disruption of DNA-binding abilities which manifest with younger age at onset due to thermodynamic destabilization and structural abnormality of the protein (100, 101). In silico analysis in combination with *in vitro* methods revealed the characteristics and functions of POU-like and a homeodomain-like motifs of HNF1α in detail. According to the results of the study (102), the substitutions located in the residues of DNA-binding domain give rise to direct (through interrupted hydrogen bonds) and indirect (through perturbed local environment) disruption of bindings between DNA and HNF1α, impaired interdomain interactions, hindered nuclear translocation, and structural instability, which in turn cause accumulation of misfolding protein. Mutations to transactivation domain destabilize interactions with co-activators or other regulatory proteins and reduce transactivation with or without diminishing DNA-binding activities (100).

Interdependency of HNF1α and HNF4α is in consistent with the similarities in the clinical phenotype and treatment option of MODY1 and MODY3 (4, 82, 91). Currently, the first-line treatment of both types is SU and/or incretin-based agents (103, 104). The exaggerated sensitivity of MODY3 patients to SUs (87) and negative role of *HNF1α* mutations on glucagon secretion and incretin levels favored the treatment with DPP-4 inhibitors (105). Comparative study is required to define the best therapeutic agent(s) in accordance with the mutational characteristics of MODY1 and MODY3, as well as *HNF1α* and *HNF4α* variants of T2DM.

### *PDX1* mutations (MODY4)

PDX1 (**Table 1**) is a homeodomain-containing transcription factor, possesses a N-terminal transactivation domain, a C-terminal domain and a DNA-binding homeodomain (HD), which are hot-spot regions for mutations (**Figure 2**) (106). Depending upon the mode of inheritance, location and penetrance of the mutation, *PDX1* gene alterations result in partial and total pancreatic agenesis (107), ND without exocrine insufficiency (108), gestational diabetes (93), and MODY with variable age at onset and severity (106). Since PDX1 is a master regulator of embryonic pancreas development, islet formation and β-cell differentiation (109), it is not surprising that its homozygous and compound heterozygous missense and frameshift mutations underlie the pancreatic agenesis (107). In the recent study, MODY4 patient-derived iPSC line was differentiated into pancreatic progenitors and genome-wide analysis of transcriptional targets of PDX1 was carried out in different stages of pancreatic cells. The analysis indicates that PDX1-binding sites for target genes evolve during embryonic development and change significantly. For instance, while PDX1 activates *HNF1α, HNF1β* and *RFX6* during early developmental stage, *KCNJ11* regulation becomes apparent only after islet formation (110). Recently, interesting case of ductal pancreatic agenesis is reported in a family carrying heterozygous frameshift mutation (111). We assume that because *PDX1* is activated by different factors in ventral and dorsal pancreatic bud (Hex1 in ventral bud, Hb9 in dorsal bud) (10), mutations may affect interaction between *PDX1* and its upstream regulators differently in an early developmental stage. Further work is needed to examine this assumption.

At least 33 MODY4 related mutations were reported so far (4, 106, 112) and their phenotypes are explained by dysregulation of glucose stimulated *INS* promoter activity at the transcriptional level by PDX-1 (113). Structural stability is vital for PDX1 in order to be capable of binding to gene promoter and the mutations to both NTD and HD are known to cause structural instability (93, 114). Reduced transcriptional activity is mostly derived from NTD mutations interfering with protein-protein interactions (93, 115). Although the function of CTD is still unclear, functional studies indicate that CTD mutations lead to diminished transactivation (115). However, according to the *in vitro* study with unnaturally generated CTD mutations, it also plays pivotal role in subnuclear localization of PDX1 through phosphorylation (116).

Management of MODY4 ranges from diet to insulin replacement depending on the degree of insulin synthesis defect. Recent studies propose DPP-4 inhibitors as a best treatment option since it is proved that *PDX1* mutations are also responsible for lower levels of incretins (106).

### *HNF1β* mutations (MODY5)

Similar to HNF1α, HNF1β (**Table 1**) has a N-terminal dimerization domain, a bipartite DNA-binding POU domain and a C-terminal transactivation domain (**Figure 2**). Its highly conserved dimerization domain and DBD permit HNF1β to be able to bind to DNA as a homodimer and heterodimer with HNF1α, while flexible transactivation domain and interdomain linkers allow it to cooperate with wide range of variable proteins in diverse tissues (117). MODY5 or renal cyst-diabetes syndrome occurs due to loss-of-function mutations and is characterized by phenotypic heterogeneity regarding the organ involvement and higher penetrance of renal anomalies than diabetes even among same family members (118). Intron 2 is the hot spot for splice-site mutations, which result in complete loss of exon 2 through yet-unidentified mechanisms and consequent truncated inactive protein (119). Most single amino acid substitutions are located in the DBD and disrupt the bonds with DNA directly or indirectly *via* perturbing local environment such as creating superfluous interactions with neighboring residues. Besides, some mutations to the same region affect protein structure and stability and leads to misfolded protein response (117).

Although the pancreatic development by HNF1β at the primitive gut stage is well demonstrated (120), the results of the studies indicating whether HNF1β is involved in GSIS and even is present or absent in mature β-cells are ambiguous (121, 122). Despite of the 70% homology in sequence identities of HNF1α and HNF1β (116), the management of MODY5 is different from MODY3. Unlike MODY3, MODY5 shows poor response to SUs and insulin replacement is the only option in majority of cases (123).

### *NEUROD1* mutations (MODY6)

With the aid of its basic helix-loop-helix and transactivation domains, NEUROD1 (**Table 1**) displays DNA-binding, dimerization and transactivation abilities (**Figure 2**) (124). NEUROD1 is highly expressed in neuroendocrine tissues and its central function in their development is evident from the mutations causing ketoacidosis-prone diabetes with microvascular sequelae and neurological abnormalities such as cerebellar hypoplasia, hearing and visual impairments, low IQ (125). Up to now, at least 26 missense and nonsense mutations found in *NEUROD1* gene, which four of them were homozygous mutations (125–129). Homozygous inheritance shows more severe phenotype, earlier onset and higher penetrance than heterozygous state (130). The analysis of the first case of MODY6 revealed that NEUROD1 heterodimerize with another bHLH (basic Helix-Loop-Helix) transcription factor – E47 and binds to E-box element of the insulin promoter to induce its synthesis through the interaction between p-300 and C-terminal glutamine-rich domain (131). Two distinct transactivation domains, namely AD1 and AD2, have different transactivation capabilities (132) and whether regulators other than p300 interacts with NEUROD1 is unclear, since studies related to the structure and function of this protein are scarce. Moreover, the role of disease-causing substitutions outside of the bHLH and C-terminal domains (132) needs to be investigated.

Treatment of MODY6 is not specific and varies from diet to insulin replacement depending upon the severity of the disease (125).

### *RFX6* mutations (Newly proposed MODY type)

RFX6 (**Table 1**) is a winged-helix transcription factor and encompasses DBD, dimerization domain D and extended dimerization domains B and C according to the homology modeling of RFX protein family (**Figure 2**) (133). Homozygous and compound heterozygous truncating mutations lead to Mitchell-Riley syndrome, the syndrome characterized with neonatal/early-onset diabetes, hypoplastic/annular pancreas, gallbladder hypoplasia/agenesis, intestinal atresia with or without intestinal malrotation (134). On the other hand, patients harboring heterozygous mutations suffer from late-onset diabetes, which show reduced penetrance (135). As an upstream regulator of *PDX-1* and *NEUROD1*, RFX6 is vital for islet formation (81). Consistent with the pancreatic anomalies observed in patients bearing *RFX6* mutations, iPSC line derived from the family suffering from Mitchell-Riley syndrome exhibited the absence of body and tail of pancreas. Besides, the study also proposed the possible regulatory loop between PDX1 and RFX6 in the pancreatic progenitor stage (136). RFX6 is able to homodimerize and heterodimerize with RFX3 to bind to X-box motif of target promoter and its role in GSIS is linked to modulatory effect on SUR1, GCK and L-type Ca^2+^ channels (84, 137). While substitutions in the conserved region of DBD completely abrogate DNA-binding, alterations in other regions partially interfere with DNA-binding abilities (81). Since RFX6 is a newly discovered protein, there is a hope that the molecular characterization of *RFX6*-MODY will provide with valuable knowledge regarding its structure and activity.

The association between *RFX6* mutations and GIP levels has been demonstrated (134). Patients with *RFX6*-MODY are responsive to treatment with DPP-4 inhibitors (138).

## MODY beyond the β-cell

### *CEL* mutations (MODY8)

**Figure 4:**
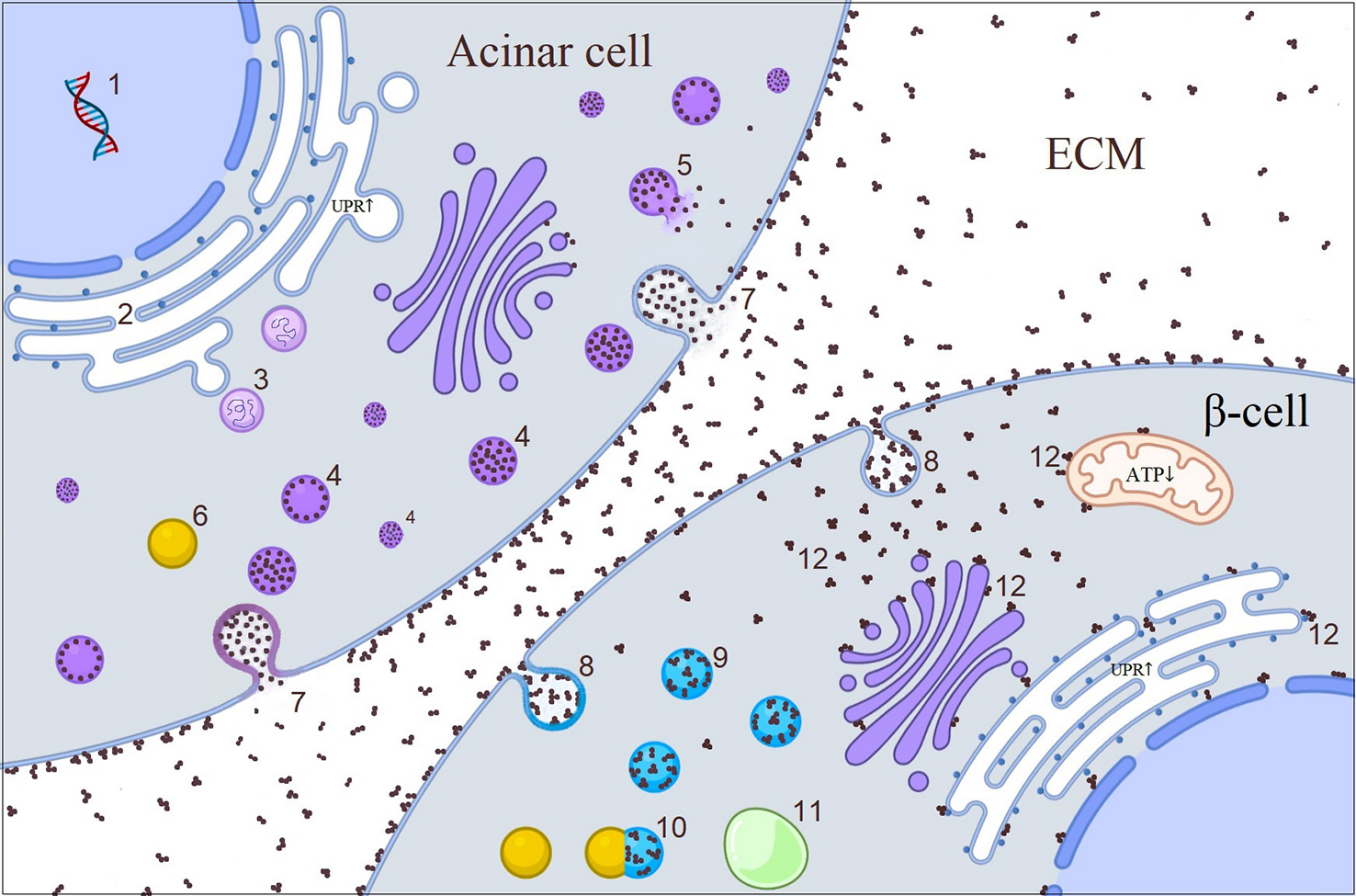
Illustrative picture of the cross-talk between acinar cell and β-cell in *CEL*-mutated pancreas. 1. CEL synthesis in acinar cell; 2. Misfolded CEL protein causing increased UPR in ER during post-translational modification; 3. UPR initiated retro-translocation of misfolded CEL protein; 4. CEL protein containing vesicles; 5. Disrupted vesicle; 6. Lysosome; 7. Exocytosis of CEL protein through either fusion of vesicles with plasma membrane or direct accumulation of the protein in the plasma membrane which later becomes core of CEL aggregates in the ECM; 8. Endocytosis of CEL aggregates; 9. Endosomes; 10. Fusion of lysosome to the endosome; 11. Autophagosome; 12. Interaction of free aggregates with the membranes (?). *CEL*, carboxyl ester lipase; *ECM*, extracellular matrix; *ER*, endoplasmic reticulum; *UPR*, unfolded protein response.

Carboxyl ester lipase (**Table 1**) is a pancreatic enzyme produced in acinar cells, but not in β-cells (139). It has signal peptide, bile-salt binding sites and catalytic sites containing globular domain, and multiple O-glycosylation sites containing polymorphic variable number tandem repeats (**Figure 2**) (140). Gain-of-function mutations to *CEL* gene give rise to pancreatic exocrine insufficiency during adolescence or young adulthood and a couple of decades later, to insulin-dependent diabetes (141). Recent few preliminary studies shed light into the impact of external CEL protein to β-cells (142–145). O-glycosylation sites at the VNTR render the CEL enzyme soluble and frameshift mutations at the VNTR turn the molecule into positively charged insoluble aggregates which is prone to bind with negatively charged structures including cell and organelle membranes (142). In acinar cells, mutant cell enzyme causes ER retention and UPR activation. In spite of acinar cells induce yet-to-be-dentified degradative machinery, the acinar cells can not overcome the excess amount of misfolded protein (144). Modified misfolded CEL protein gain access to extracellular space through induction of exocytosis and then forms insoluble aggregates (143). Cross-talk between acinar and islet cells allows the insoluble aggregates to be re-uptaken by neighboring β-cells *via* endocytosis (145). Eventually, the capability of β-cells to degrade endocytic substrates through lysosomal pathways depletes (145). We assume that “sticky” CEL aggregates interact with the membranes inside the cell and this in turn triggers dysfunction of different organelles displayed as altered mitochondrial activity and ER stress (**Figure 4**) (143). In fact, direct interaction of another toxic misfolded protein product – Islet Amyloid Polypeptide Oligomers with the membranes in β-cells had been demonstrated by electron microscopy (146). On the other hand, other possible toxic pathways such as expression of “disallowed” genes or β-cell-senescence can not be ruled out (145). However, structural characteristics of CEL aggregates, mechanism of exocytosis and endocytosis, behavior of internalized macromolecules within β-cells and involved pathways are largely unknown and further *in vitro* studies using variety of approaches are highly demanded, which may also have therapeutic implications.

## Summary

Monogenic diabetes gave us simplified models of complex molecular processes occurring within β-cells, which allowed to explore the roles of numerous proteins from single protein perspective. Clarification of the structural and functional abnormality of a particular protein, which arose from a mutation to its gene, helps to define the crystal structure, function and interaction with other molecules of this protein. The characteristics of the mutations also help to identify phenotypical heterogeneity and have implications for personalized medicine. Most importantly, all of these enlightenments pave the way for the new treatment strategies. In fact, understanding of the kinetic parameters of GCK enzyme brought in new antidiabetic agents which have potential to fulfill the need for an optimum drug to control glucose levels efficiently with minimum side effects in the management of T2DM (27). K_ATP_ channel is the target for SUs and switching therapy to SUs free the patients from the burden of insulin therapy (54, 55). SUs are also effective in the management of MODY1 and MODY3, probably due to regulation of K_ATP_ channel subunits by HNF1α (87, 104). On the other hand, insulin requirement in almost all cases of MODY5 might be explained by the central role of *HNF1β* in the early pancreatic development rather than its involvement in glucose homeostasis (123). Clinically observed association between GLP-1/GIP and some MODY-related genes (at least *PDX1* and *RFX6*), of which molecular basis is unknown, made the treatment with GLP-1 analogs or DPP-4 inhibitors to be favorable (106, 134). *INS*, *WFS1* and *CEL* mutations that lead to ER retention and UPR (61, 70, 145), might be rescued by therapies targeting these toxic pathways. Moreover, extrapolation of data related to the aforementioned transcription factors into the stem cell research has potential to provide the radical treatment for T1DM patients.

In the last century, the approach to MODY was based on the investigation of the previously known proteins (147) and this approach broadened our comprehension regarding their role in developing and mature β-cells. The application of sequencing methods to experimental and clinical medicine (148) introduced new molecules involved in β-cell development, identity, and function. Extensive exploration of these proteins not only resulted in better medical care to diabetes patients, but also enriched our understanding about the universal cellular processes including transcriptional and translational regulation, behavior of ion channels and transporters, cargo trafficking, exocytosis. Noteworthy, β-cells are important model to test similar events in other cells. In the recent years, state-of-the-art technique – the establishment of MODY patient-derived stem cell lines – has been added to ongoing diabetes research and is expected to yield breakthrough treatment options in the near future, of which benefits might exceed diabetes management.

## Author contributions

AZ provided the first draft of the manuscript. WG and BZ carried out literature search and designed the table and the figures. SS and QZ wrote the manuscript. All authors revised and approved the final version of the manuscript.
